# Maternal postnatal depression and anxiety and the risk for mental health disorders in adolescent offspring: Findings from the Avon Longitudinal Study of Parents and Children cohort

**DOI:** 10.1177/00048674221082519

**Published:** 2022-03-02

**Authors:** Isabel Morales-Munoz, Brooklyn Ashdown-Doel, Emily Beazley, Camilla Carr, Cristina Preece, Steven Marwaha

**Affiliations:** 1 Institute for Mental Health, School of Psychology, University of Birmingham, Birmingham, UK; 2 Department of Public Health Solutions, Finnish Institute for Health and Welfare (THL), Helsinki, Finland; 3 Specialist Mood Disorders Clinic, Birmingham and Solihull Mental Health NHS Trust, Birmingham, UK

**Keywords:** Avon Longitudinal Study of Parents and Children, postnatal depression, postnatal anxiety, adolescence, mental health

## Abstract

**Objective:**

The impacts of postnatal psychiatric disorders on different types of mental health problems in offspring are unclear. We investigated the prospective associations of maternal postnatal depression, and anxiety, with offspring depression, anxiety, psychotic-like experiences and Borderline Personality Disorder symptoms, in adolescence, and examined whether these were independent of each other.

**Methods:**

Data were obtained from the Avon Longitudinal Study of Parents and Children (ALSPAC) birth cohort. Maternal postnatal depression and anxiety at 8 weeks were measured using the Edinburgh Postnatal Depression Scale and Crown-Crisp Index, respectively. Offspring mental health outcomes were measured at 10–13 years old, using a variety of questionnaire-based and interview assessments. Logistic regression analyses were used to assess the associations between maternal postnatal risk factors and offspring mental health, and path analysis was used to investigate the pathways of maternal postnatal factors to adolescent offspring outcomes.

**Results:**

Data were available for 14,054 mothers with information reported on postnatal depression and 13,892 on postnatal anxiety. Logistic regression analyses found significant associations between maternal postnatal depression and offspring anxiety at 10 years old (odds ratio = 1.039, 95% confidence interval = [1.005, 1.073], *p* = 0.022) and between maternal postnatal anxiety and offspring psychotic experiences at 12/13 years old (odds ratio = 1.042, 95% confidence interval = [1.008, 1.077], *p* = 0.016). These significant associations remained after applying path analyses, when we controlled for potential offspring psychopathological overlay.

**Conclusion:**

These findings suggest that mothers with postnatal depression are more likely to have offspring with anxiety at 10 years old, and that mothers with postnatal anxiety are more likely to have offspring with psychotic experiences at 12/13 years old. Our findings suggest specific pathways in the association between postnatal anxiety/depression and offspring mental health and contribute to the importance of identifying mothers and their offspring with increased vulnerability to adverse outcomes resulting from postnatal mental health disorders.

## Introduction

The perinatal period represents a period of increased vulnerability for women, characterized by risk for maternal mental health problems, and this period occurs during pregnancy and the first year following the childbirth (Cristescu et al., 2015). Therefore, the perinatal period refers to pregnancy (i.e. antenatal) and postpartum (postnatal). Perinatal mental health problems comprise a spectrum of pre-existing and novel conditions, including depression and anxiety which affects approximately 13% of women in high-income countries and 15–20% in low-/middle-income countries (Collins et al., 2011).

The postnatal period, which is the focus of this study, is one of the most crucial times in the lives of mothers and their babies. This period is important because approximately 60% of maternal death occurs during this period, it is a crucial time for establishing breastfeeding, and importantly, it is a period when mothers are highly vulnerable to various mental health problems (Rai et al., 2015). Postnatal mental health problems often adversely impact offspring developmental trajectories over time, including physical health, sleep, motor, cognitive and/or socio-emotional development, and/or behaviour. For example, postpartum depression may reflect chronic depression, potentially affecting offspring neurodevelopment and increasing the risk of poor offspring mental health (Grace et al., 2003). Furthermore, postnatal anxiety has negative effects on infant temperament, sleep, mental development, health and internalizing behaviour and/or conduct disorder in adolescents (Field, 2018).

Postnatal maternal depression is the most frequently investigated postnatal risk factor for mothers and offspring mental health problems. It affects approximately 13% of mothers (Leahy-Warren and McCarthy, 2007). Postnatal depression not only has negative consequences for the mothers’ well-being, but also is associated with an increased risk of adverse developmental outcomes for offspring, including behavioural difficulties, impaired cognitive ability and/or persistent internalizing and externalizing behaviours (Grace et al., 2003). More specifically, maternal postnatal depression is a strong predictor for adolescent offspring depression (Pearson et al., 2013). For instance, offspring of postnatally depressed mothers are at increased risk for depression by 16 years of age (Murray et al., 2011). In addition, maternal postnatal depression is associated with offspring persistent depression in childhood and early-adult-onset depression (Kwong et al., 2019). However, the impact of postnatal depression is not limited to offspring depression, and it extends to other affective symptoms, including anxiety. For instance, maternal postnatal depression is associated with higher rates of anxiety disorders in adolescent offspring (Halligan et al., 2007).

The impact of maternal anxiety upon offspring development and mental health has received less attention compared to maternal depression. Existing studies have focused on the effect that postnatal anxiety exerts on offspring development in childhood but not in adolescence, with previous research reporting that postnatal anxiety is linked to maternal reports of children emotional problems at 4 years old (O’Connor et al., 2002b) and at 6 years old (O’Connor et al., 2003). Therefore, further research investigating the impact of postnatal anxiety on offspring mental health is required, and especially during adolescence.

Finally, there are other mental health problems in offspring that could be also impacted by maternal postnatal depression and/or anxiety, but which have received little attention. More specifically, the links of postnatal depression and anxiety, with psychosis and/or Borderline Personality Disorder (BPD) have received relatively little attention to date, although postnatal experiences could be an important factor in helping explain both conditions. For example, maternal postnatal depression negatively affects bonding and parenting during infancy, which might affect offspring attachment style (Stein et al., 2014) and increase the risk of psychotic experiences. Furthermore, attachment problems, which could arise as a consequence of maternal postnatal mental disorders, are an especially relevant factor to consider in BPD (Agrawal et al., 2004). In relation to psychosis, the impact of maternal postnatal depression has been recently investigated in relation to offspring psychosis. Evidence supported an association between maternal perinatal (i.e. antenatal and postnatal) depression and offspring psychotic experiences at 18 years old, using a longitudinal UK birth cohort (Srinivasan et al., 2020).

However, the impact of postnatal depression on offspring psychosis remains critically under-researched, with limited understanding especially during adolescence. Adolescence is a key developmental period to study the onset of several mental disorders, because of brain and hormonal changes occurring during this period (Blakemore et al., 2010). Therefore, further research investigating the impact of early risk factors (e.g. postnatal factors) on adolescents’ mental health is needed. In relation to psychosis, there is evidence for the existence of adolescent-specific developmental mechanisms that impart vulnerability to psychosis in adolescence (Patel et al., 2021).

In relation to BPD, this is a disorder that has special importance in adolescence, with theory and empirical research pointing to adolescence as a key developmental period for the onset of BPD (Wright et al., 2016). Therefore, understanding the impact that postnatal factors might exert in the development of BPD symptoms in adolescence would shed light on potential early life risk factors for this condition. The scarce existing research has focused on the impact of antenatal depression and/or anxiety, rather than postnatally, but results are often inconsistent. While there is some evidence to support the hypothesis that prenatal anxiety and depression are independently associated with BPD in early adolescence (Winsper et al., 2015), a recent study reported that the sons of antenatally depressed mothers had an increased risk for antisocial personality disorder but not for BPD (Taka-Eilola et al., 2020).

To our knowledge, the existing research investigating the associations between maternal postnatal depression and/or anxiety and offspring mental health in adolescence has been limited to one or a few mental health areas. To what extent maternal postnatal depression and/or anxiety precede a range of mental health disorders in the same sample is unknown. In other words, to date, there is little evidence on the extent to which these exposures are important in independently explaining multiple mental health outcomes. Mood disorders, anxiety, personality disorders and/or psychosis all most commonly emerge during adolescence, and thus, it is important to disentangle which early risk factors, including maternal postnatal factors, are more likely to precede each of these mental health problems. It is essential to do so to understand how mental health disorders develop, whether there are individual pathways associated with the development of specific mental health disorders, and to what extent this development could be changed.

Therefore, to address this gap in the literature, we aimed to investigate the prospective associations of maternal postnatal depression and anxiety, with offspring depression, anxiety, psychotic experiences and BPD symptoms, in adolescence. Furthermore, we aimed to investigate maternal postnatal depression and anxiety simultaneously due to their high comorbidity and large symptom overlap. Despite high comorbidity between depression and anxiety during the postnatal period, the two are rarely investigated simultaneously. We hypothesized that when simultaneously investigating the impact of maternal postnatal depression and anxiety, both would be associated with offspring depression, psychotic experiences, BPD symptoms and anxiety in adolescence.

## Methods

### Participants

This study used data from the Avon Longitudinal Study of Parents and Children (ALSPAC), a UK birth cohort that examines the factors associated with development, health and disease during childhood and beyond (Boyd et al., 2013). Pregnant women with expected dates of delivery from 1 April 1991 to 31 December 1992 were invited to take part in the study. The ALSPAC website contains details of all the data available through a fully searchable data dictionary and variable search tool (www.bristol.ac.uk/alspac/researchers/our-data/). The initial number of pregnancies enrolled was 14,541 (for these at least one questionnaire was returned or a ‘Children in Focus’ clinic had been attended by 19 September 1999). Of these initial pregnancies, there were a total of 14,676 foetuses, resulting in 14,062 live births and 13,988 children who were alive at 1 year. When the oldest children were approximately 7 years old, an attempt was made to bolster the initial sample with eligible cases who had failed to join the study originally. As a result, in this study, as some variables were collected from the age of 7 onwards, there were data available for more than the 14,541 pregnancies mentioned above. The number of new pregnancies not in the initial sample (known as Phase I enrolment) that are currently represented on the built files and reflecting enrolment status at the age of 24 years is 913 (456, 262 and 195 recruited during Phases II, III and IV, respectively), resulting in an additional 913 children being enrolled. The total sample size for analyses using any data collected after the age of 7 is therefore 15,454 pregnancies, resulting in 15,589 foetuses. Of these, 14,901 were alive at 1 year. Ethical approval was obtained from the ALSPAC law and ethics committee and the local research ethics committees. Informed consent for the use of data collected via questionnaires and clinics was obtained from the participants following the recommendations of the ALSPAC Ethics and Law Committee at the time.

### Measures

#### Maternal postnatal depression at 8 weeks old

The Edinburgh Postnatal Depression Scale (EPDS) is a 10-item self-report questionnaire to measure depressive symptom severity. This scale is the most widely used screening tool in the pre- and postnatal period, with evidence for its use in the postnatal period (Gibson et al., 2009). Items are scored based on how the mother feels over the past 7 days. The psychometric properties of the EPDS are 86% sensitivity, 78% specificity and 73% positive predictive value, and good internal consistency at a level of 0.83 has been reported (Bunevicius et al., 2009). For this study, we used the total score.

#### Maternal postnatal anxiety at 8 weeks old

The Crown-Crisp Index (CCEI) is a validated self-rating inventory, and measures six different types of neurotic traits and symptoms. The CCEI presents strong correlations (i.e. 0.70 and 0.76) with the Spielberger State-Trait Anxiety Inventory, and has demonstrated internal consistencies of >0.80 across assessments (Heron et al., 2004). For this study, the total score from the anxiety subscale was used, which measures the presence of anxiety symptoms using eight items.

#### Offspring psychotic experiences at 12/13 years old

The Psychosis-Like Symptom Interview (PLIKSi) is a semi-structured interview consisting of 12 core questions derived from the Diagnostic Interview Schedule for Children Version IV (DISC-IV) and Schedules for Clinical Assessment in Neuropsychiatry (SCAN). The interviewers were psychology graduates trained in the use of the SCAN psychosis section and the PLIKSi. The questions are designed to assess the presence of delusions, hallucinations and thought interference. Offspring were asked if they had ever experienced any of these symptoms from the age of 12 years old. Interviewers rated experiences as ‘not present’, ‘suspected’ or ‘definitely psychotic’. The average kappa value for inter-rater reliability of rating psychotic experiences is 0.83 and the kappa for test–retest reliability is 0.76 (Zammit et al., 2013). For this study, we coded the presence of at least one definite psychotic symptom not attributable to sleep or fever (Zammit et al., 2013).

#### Offspring depression at 10 years old

The Mood and Feelings Questionnaire is a 13-item self-reported questionnaire to assess the experience of depressive symptoms over the past 2 years. There is evidence reporting good internal consistency, with Cronbach’s alpha values ranging from 0.91 to 0.95 (Daviss et al., 2006). A cut-off score of ⩾11 was used, due to high sensitivity and specificity for major depression (Eyre et al., 2021).

#### Offspring anxiety at 10 years old

Parents completed the Development and Well-Being Assessment (DAWBA) in relation to their offspring. This assessment consists of questionnaires, interviews and rating techniques which are designed to produce psychiatric diagnoses according to the *International Statistical Classification of Diseases and Related Health Problems–Tenth Edition* (ICD-10) and the *Diagnostic and Statistical Manual of Mental Disorders* (4th ed.; DSM-IV) criteria. The initial validation study of the DAWBA suggests that it has considerable potential as an epidemiological measure and as a clinic assessment (Goodman et al., 2000). The presence of any anxiety disorder was coded according to these diagnostic criteria (Goodman et al., 2000).

#### Offspring BPD symptoms at 11/12 years old

BPD symptoms were assessed using the UK Childhood Interview for DSM-IV Borderline Personality Disorder (UK-CI-BPD), a face-to-face semi-structured interview based on the borderline module of the Diagnostic Interview for DSM-IV Personality Disorders. The inter-rater and test–retest reliability of the *Diagnostic and Statistical Manual of Mental Disorders* (3th ed.; DSM-III), *Diagnostic and Statistical Manual of Mental Disorders* (3th ed., rev.; DSM-III-R) and DSM-IV versions of this measure have all proven to be good to excellent in this respect (Zanarini and Frankenburg, 2001). The outcome was dichotomized according to the frequent or repeated occurrence of ⩾5 BPD symptoms (Winsper et al., 2020).

#### Covariates

Multiple family risk factors were assessed using the Family Adversity Index (FAI) during pregnancy (long index), at 2 years (long index) and at 4 years (short index). FAI comprises 18 items (i.e. long index) on early parenthood, housing conditions, maternal education, financial difficulties, parents’ relationship, family size, family major problems, maternal psychopathology, parental substance abuse, crime records, partner support and social network. Points were summed at each time point for a total FAI score. We included this variable as confounder, as early adversity is a well-established risk factor for poor mental health outcomes in adolescents (Wadman et al., 2020).

Child sex, child ethnicity, weight (in kg) at birth, gestational age (in weeks) and maternal age at birth were also included as covariates. Child ethnicity was dichotomized into ‘white’ and ‘other’.

### Statistical analysis

All statistical analyses were conducted using SPSS-v27 (SPSS Inc., Chicago, IL, USA). First, a descriptive analysis for all variables of interest was conducted. Furthermore, correlations between both predictors and all the covariates were conducted to assess the strength of the relationship between these variables.

For the main analysis, a series of logistic regression analyses were conducted, to assess the associations of maternal postnatal depression and anxiety with offspring psychotic experiences (at 12/13 years old), depression (at 10 years old), anxiety (at 10 years old) and BPD symptoms (at 11/12 years old). First, to deal with missingness, we conducted logistic regressions to identify significant factors associated with attrition. The individuals associated with attrition in early adolescence had younger mothers when the baby was born, shorter gestational age, weighed less at birth, reported higher family adversity problems and were more frequently non-White. Using the variables associated with selective dropout as the factors, we fitted a logistic regression model (non-response vs response outcome) to determine weights for each individual using the inverse probability of response. The regression coefficients from this model were used to determine probability weights for the covariates in the main analyses.

For the logistic regression analyses, both predictors and all the covariates were included together, while separate analyses were conducted for each outcome. First, unadjusted models were applied, and second, we conducted adjusted logistic regression models, controlling for all covariates mentioned above. Both predictors (postnatal depression and anxiety) were included together in the same step, due to (1) a moderate correlation between the two variables (*r* = 0.578) and (2) our initial interest in controlling for the effect of each other.

Finally, path analysis was conducted using SPSS Amos-v27 (SPSS Inc.), to investigate the pathways of maternal postnatal depression and anxiety to different mental health outcomes in the offspring, while controlling for any interactions between the outcome variables. The predictor variables included in the model were maternal postnatal depression and anxiety, and the outcomes were offspring psychotic experiences, depression, anxiety and BPD symptoms in adolescence. In this path analysis, we tested the significant associations obtained from the logistic regression analyses. Furthermore, FAI, gender and gestational age were included as covariates. These were selected based on their significant associations in the adjusted logistic regression model. The associations between FAI and all the outcomes are well tested in previous literature. Missing data were dealt with using the full information maximum likelihood method.

## Results

### Descriptive results

Among the initial sample of 14,741 individuals, data were available for 14,054 mothers with information on postnatal depression and 13,892 mothers with information on postnatal anxiety. Among the offspring outcome measures, data were available for 9212 individuals with information on depression at 10 years old, 9893 individuals reported information on anxiety at 10 years old, data were available for 8233 individuals with information on psychosis at 12/13 years old, and finally for BPD, data were available for 7127 individuals at 11/12 years old (see Table 1 for all the descriptive values).

**Table 1.** Descriptive characteristics of independent variables, outcomes and covariates included in the study.

Variable	*N*	%	Minimum	Maximum	Mean	SD
Gender (*n* = 14,741)						
Male	7609	51.6				
Female	7132	48.4				
Birth weight (kg) (*n* = 14,741)			0.65	5.64	3.42	0.54
Gestational age (weeks) (*n* = 14,741)			25	47	39.47	1.82
Mother age at delivery (years) (*n* = 14,741)			15	44	28.1	4.82
Childs race/ethnicity (*n* = 14,741)						
White British	14,390	97.6				
Other ethnic group	351	2.4				
FAI total score (*n* = 14,741)			0	31	4.98	4.8
Maternal depression total score at 8 weeks postpartum^a^ (*n* = 14,054)			0	28	6.13	4.82
Maternal anxiety total score at 8 weeks postpartum^b^ (*n* = 13,892)			0	16	3.73	3.42
Offspring depression at 10 years^c^ (*n* = 9212)						
Yes	575	6.2				
No	8637	93.8				
Offspring anxiety disorder at 10 years^d^ (*n* = 9893)						
Yes	217	2.2				
No	9676	97.8				
Offspring BPD symptoms at 11/12 years^e^ (*n* = 7127)						
Yes	657	7.3				
No	7160	92.7				
Offspring psychosis symptoms at 12/13 years^f^ (*n* = 8233)						
Yes	467	5.7				
No	7766	94.3				

SD: standard deviation; FAI: Family Adversity Index; BPD: Borderline Personality Disorder.

a Measured with the Edinburgh Postnatal Depression Score (EPDS).

b Measured with the Crown-Crisp Experiential Index (CCEI).

c Measured with the Short Mood and Feelings Questionnaire (SMFQ).

d Measured with the Development and Well-Being Assessment (DAWBA).

e Measured with the Childhood Interview for DSM-IV BPD identification.

f Measured with the Psychosis-Like Symptom Semi-Structured Interview (PLIKSi).

### Correlation analysis

The two-tailed Pearson correlations between the independent variables and the covariates are displayed in Table 2. Significant results to the 0.01 level were shown for most of the associations. The strongest association was found between both predictors (*r* = 0.578), followed by the associations between each predictor with FAI (maternal postnatal depression: *r* = 0.424; maternal postnatal anxiety: *r* = 0.425).

**Table 2.** Correlation analysis results between the independent variables and covariates.

Variable	Gender	Birth weight	Gestational age	Mother’s age at delivery	Childs Ethnicity	Family Adversity Index	Maternal postnatal depression	Maternal postnatal anxiety
Maternal postnatal depression	−0.002	−0.043*	−0.061*	−0.036*	0.053*	0.424*	−	0.578*
Maternal postnatal anxiety	0.006	−0.034*	−0.032*	−0.006	0.032*	0.425*	0.578*	−

* Correlation is significant at the 0.01 level.

### Logistic regression analysis

Table 3 shows the prospective associations between maternal postnatal mental health problems and adolescent offspring mental health outcomes. The unadjusted model showed significant associations for both postnatal anxiety and depression with all the outcomes (BPD, depression, anxiety and psychosis), except for postnatal depression and offspring BPD, where no significant associations were found. After adjusting for all the covariates, the only significant associations that remained were maternal postnatal depression with offspring anxiety at 10 years old, and maternal postnatal anxiety and offspring psychotic experiences at 12/13 years old. Furthermore, among the covariates, FAI was associated with all the outcomes (see Supplementary Material Table S1, for statistical values for all the covariates).

**Table 3.** Logistic regression analyses between maternal postnatal depression and anxiety, and adolescent offspring mental health outcomes.

	Unadjusted model				Adjusted model*			
	β	SE	*p*	OR [95% CI]	β	SE	*p*	OR [95% CI]
Offspring BPD symptoms at 11/12 years old								
Postnatal depression at 8 weeks postbirth	0.012	0.012	0.289	1.012 [0.990, 1.036]	0.001	0.012	0.940	1.001 [0.978, 1.025]
Postnatal anxiety at 8 weeks postbirth	**0.050**	**0.015**	<0.001**	**1.052 [1.021, 1.084]**	0.028	0.016	0.079	1.028 [0.997, 1.061]
Offspring depression at 10 years old								
Postnatal depression at 8 weeks postbirth	**0.024**	**0.011**	0.033**	**1.024 [1.002, 1.046]**	0.003	0.011	0.810	1.003 [0.981, 1.025]
Postnatal anxiety at 8 weeks postbirth	**0.059**	**0.015**	<0.001**	**1.061 [1.031, 1.092]**	0.029	0.015	0.063	1.029 [0.998, 1.061]
Offspring anxiety at 10 years old								
Postnatal depression at 8 weeks postbirth	**0.062**	**0.016**	<0.001**	**1.064 [1.031, 1.098]**	**0.038**	**0.017**	0.022**	**1.039 [1.005, 1.073]**
Postnatal anxiety at 8 weeks postbirth	**0.053**	**0.022**	0.018**	**1.054 [1.009, 1.102]**	0.024	0.023	0.289	1.025 [0.979, 1.072]
Offspring psychotic experiences at 12/13 years old								
Postnatal depression at 8 weeks postbirth	**0.025**	**0.012**	0.037**	**1.026 [1.002, 1.050]**	0.015	0.012	0.225	1.015 [0.991, 1.040]
Postnatal anxiety at 8 weeks postbirth	**0.062**	**0.016**	<0.001**	**1.064 [1.031, 1.098]**	**0.041**	**0.017**	0.016**	**1.042 [1.008, 1.077]**

BPD: Borderline Personality Disorder; SE: standard error; OR: odds ratio; CI: confidence interval; *p*: statistical significance; β: unstandardized beta.

We included both postnatal depression at 8 weeks postbirth and postnatal anxiety at 8 weeks postbirth within the same model.

See Supplementary Material Table S1, for statistical values for each of the covariates.

* Covariates: offspring birth weight (kg), offspring gender, age of mother at delivery (years), offspring ethnicity, total Family Adversity Score and gestational age of offspring at birth.

** Significant results. Bold is significant at the 0.05 level.

### Path analysis

**Figure 1.**
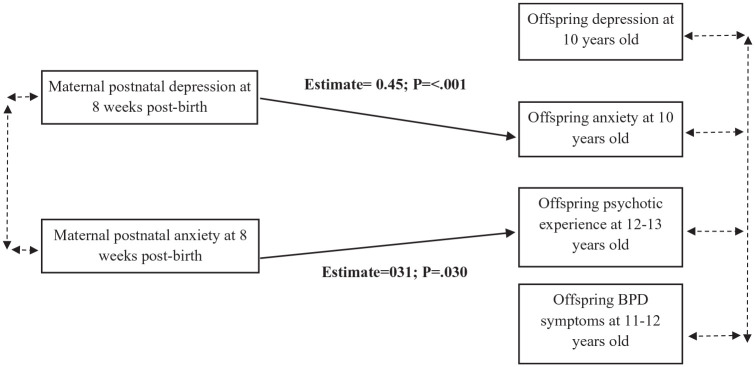
Path diagram of direct associations in the adjusted model. BPD: borderline personality disorder. This figure represents the direct associations between the two predictors (maternal postnatal depression and maternal postnatal anxiety) and the four outcomes (offspring depression at 10 years, offspring anxiety at 10 years, offspring psychotic experiences at 12/13 years and offspring BPD symptoms at 11/12 years). Arrowheads indicate the direction of the association, solid single-headed arrows indicate the significant associations, and dashed double-headed arrows indicate the controlled pathways. Covariates (not shown) included in the path analysis were offspring gender, gestational age and total family adversity index score, as these were the covariates which showed the greatest associations in the logistic regression models. Significant path associations were also reported for family adversity index and all the adolescent offspring mental health outcomes separately (all *p* < 0.001 which reflects the associations concluded in the logistic regression model. associations concluded in the logistic regression model), but they were not included in Figure 1 to ensure clarity of the model. See Supplementary Material Table S2, for statistical values for the direct effect between all the exposures, including covariates, and the outcomes.

The path analysis model fit indices suggested good model fit (χ^2^ = 16.13; *p* = 0.306; root mean square error of approximation = 003; comparative fit index = 1.00). The path analysis results were consistent with the results from the logistic regression adjusted model, showing that maternal postnatal depression was associated with offspring anxiety at 10 years old, and maternal postnatal anxiety was associated with offspring psychotic experiences at 12/13 years old, after controlling for the potential overlap of adolescent psychiatric symptoms. Significant path associations were also found between FAI and all the outcomes (all *p* < 0.001; see Supplementary Material Table S2, for statistical values for all the covariates). Direct associations are shown in Figure 1.

## Discussion

### Principal findings and comparisons to previous research

Our main findings show association between maternal postnatal depression and offspring anxiety at 10 years old, and between postnatal maternal anxiety and offspring psychotic experiences at 12/13 years old. Importantly, these associations remained after controlling for the potential symptom overlap within adolescent psychopathology. Contrary to our hypothesis, we did not find postnatal depression and/or postnatal anxiety being associated with offspring depression at 10 years old, or with offspring BPD symptoms at 11/12 years. There was also no association between postnatal depression and offspring psychotic experiences at 12/13 years old, which was also inconsistent with our hypotheses.

The consequences of postnatal depression on child development are well-established, with previous studies supporting an intergenerational transmission of depression from mothers to offspring (Hammen et al., 2012). Furthermore, maternal postnatal depression is associated with behavioural difficulties, impaired cognitive ability and decreased emotional functioning, and this is not only restricted to infancy, but also extends into toddlerhood, preschool and school age, adolescence and/or young adulthood (Canadian Pediatric Association, 2004). The findings from this study indicated that postnatal depression was associated with offspring anxiety at 10 years old. This is supported by previous research reporting a strong association between postnatal depression and elevated risk for emotional problems, such as anxiety in the offspring in adolescence (Halligan et al., 2007).

We did not find any association between postnatal depression and offspring depression, with this being dissimilar to previous findings (Gutierrez-Galve et al., 2019). A potential explanation for the inconsistency of these findings could be the timepoint at which we examined offspring depression. Most prior evidence has reported associations in later adolescence, whereas we explored offspring depression at 10 years old (i.e. early adolescence). Therefore, this suggests that depression symptomology may not present initially, but may show an increased risk along the adolescent time period. For instance, the prevalence of depression in children is low (<1%), and then rises substantially throughout adolescence (Costello et al., 1996). Another explanation for this discrepancy could be the use of the EPDS for assessing maternal postnatal depression as opposed to a diagnostic measure of depression. For example, previous research has reported maternal postnatal depression being associated with offspring psychopathology at 11 years old when using a diagnostic instrument, but not when using the EPDS (Pawlby et al., 2008).

Compared to postnatal depression, less is known about the impact of postnatal anxiety on offspring psychopathology. The existing research has reported associations between postnatal anxiety and offspring emotional problems in childhood (O’Connor et al., 2002b, 2003), but not in adolescence (Glasheen et al., 2013), and also between postnatal anxiety and conduct disorders at 16 years old (Glasheen et al., 2013). This is the first study to report a significant association between maternal postnatal anxiety and offspring psychotic experience in adolescence. Our findings are partially supported by previous research reporting a contribution of maternal stress during pregnancy to the development of a vulnerability to psychosis and schizophrenia in offspring (Abel et al., 2014), but further research that specifically investigates the impact of maternal postnatal anxiety on psychosis is required. Contrary to our hypothesis, postnatal anxiety was not associated with offspring anxiety at 10 years. So far, only three studies have reported significant associations between postnatal anxiety and offspring anxiety (O’Connor et al., 2002a, 2002b, 2003), but in younger participants (i.e. 4 and 6.75 years) than in this study (i.e. 10 years). The lack of association between maternal postnatal anxiety and offspring anxiety disorder in adolescence in this study is consistent with the results of Glasheen et al. (2013), who found that pre- and postnatal anxieties were not associated with any anxiety disorder at 16 years old. Therefore, it seems that postnatal anxiety might impact offspring anxiety in childhood, but not in adolescence. However, the lack of literature on maternal postnatal anxiety and subsequent anxiety in adolescent offspring means that further investigation of this area is needed.

There are several mechanisms that may explain our findings. First, theoretical models have proposed genetic effects for intergenerational transmission including the influence of shared genes and inherited genotype (Stein et al., 2014). There is also evidence that if a mother is depressed in the antenatal period, which is a major risk factor for postnatal depression, placental function may be altered due to increased cortisol and serotonin levels potentially influencing foetal development (O’Donnell and Glover, 2016). Depression is also associated with over activity in the hypothalamic–pituitary–adrenal axis, producing a consequent secretion of glucocorticoids, which signify a neuroendocrine response to stress (Pariante, 2003). This would consequently lead to elevated foetal levels in depressed mothers, which would potentially affect offspring neurodevelopment. This disruption in foetal development linked to maternal postnatal depression may therefore contribute to adolescent offspring anxiety development. The association between postnatal anxiety and offspring psychotic experiences at 12/13 years old may be also explained by perinatal stress due to potential development of the psychosis-associated foetal dopaminergic sensitivity mechanism (Di Forti et al., 2007) or the neurodevelopmental hypothesis of schizophrenia (Owen et al., 2011). Both suggest that abnormal foetal development affected by maternal stress may increase the risk of offspring psychotic experiences.

Second, postnatal maternal psychopathology and subsequent offspring mental health disorders may be mediated by the postnatal environment. One potential mediator is the type of attachment between the mother and the infant. Previous research suggests that insecure attachment to the mother in infancy, which is associated with postnatal depression (Martins and Gaffan, 2000), might confer psychological vulnerability in the offspring. For instance, offspring of postnatally depressed mothers are at increased risk for depression in adolescence, and this may be partially explained by the exposure to insecure infant attachment (Murray et al., 2015). Another postnatal environmental factor that might explain the associations between maternal postnatal psychopathology and offspring mental health problems is parenting. For instance, there is evidence for a significant link between postnatal mental health disorders and disruptive parenting behaviours, which subsequently may limit parents’ emotional availability, which would lead to problems in the infants’ emotional development (Aktar et al., 2019). In this study, we found that family adversity was significantly associated with all the mental health outcomes, supporting the impact that negative family environment exerts on the offspring mental health (Wadman et al., 2020).

The prevalence of offspring mental health outcomes in adolescence helps to frame our findings. We found significant associations between maternal postnatal psychopathology with anxiety and psychotic experiences, but not with depression and/or BPD symptoms. Anxiety and psychotic experiences have a higher prevalence in adolescence than depression and/or BPD, with a prevalence of 24.9% for anxiety disorders in adolescence (Kessler et al., 2012) and a prevalence for psychotic experiences of around 17% among individuals aged 9–12 years (Kelleher et al., 2012). However, estimates of prevalence of depression among children and adolescents in the community range from 2% to 6% (Costello et al., 1996), and the prevalence of BPD in the general population of adolescents is around 3% (Guilé et al., 2018). Overall, the prevalence of mental health disorders is high in adolescence, and our findings help elucidate a risk factor for this.

It is important to differentiate between mental health symptoms and disorders, with mental health symptoms being more frequent and prevalent than the disorders. For instance, studies indicate that 20–25% of adolescents experience symptoms of emotional distress and about 10% have moderate to severe symptomatology, indicating a significant impairment (SAMHSA, 2008). Therefore, the impact of postnatal risk factors would be expected to be greater on the symptomatology rather than on the disorder. However, this study was not designed to compare absolute risk of disorder in people exposed vs unexposed. Also, and in relation to psychosis, it is important to highlight that in this study, we focused on psychotic experiences and not on schizophrenia. So far, only one study has recently investigated the association between maternal perinatal depression and offspring psychotic experiences (Srinivasan et al., 2020), and reported that both antenatally and postnatally maternal depression associated with offspring psychotic experience at age 18 years. Furthermore, our findings concerning the association between postnatal anxiety and offspring psychotic experiences cannot be generalized to schizophrenia, although a small number of those impacted by psychotic experiences in adolescence do go on to develop schizophrenia. Therefore, future studies should specifically investigate the impact of maternal postnatal psychopathology on offspring schizophrenia later in life, or on whether postnatal depression and anxiety could increase this rate of conversion from psychotic experience to a diagnosis of schizophrenia.

### Strengths

This study has several strengths. First, the data used are based on a large and representative cohort study. Second, we included maternal postnatal anxiety as well as maternal postnatal depression in our analysis, alongside more under-investigated mental health outcomes in adolescence, such as BPD symptoms and psychotic experiences. Third, both predictors (i.e. maternal postnatal depression and anxiety) have only been minimally examined together, mainly regarding adolescent anxiety and depression.

### Limitations

First, although we were able to control for some relevant confounders such as family adversity, we were unable to control for other potentially relevant factors such as obstetric complications. Second, the majority of the individuals were white British; thus, our results may not account for ethnic variations. Third, this study included offspring outcome at a single time point only, and thus, we cannot comment on the longitudinal trajectories of adolescent mental health. Fourth, as might be expected in a longitudinal birth cohort study, the attrition rate was significant. However, we used procedures to ensure the representativeness of our results. Fifth, although our longitudinal study design cannot determine causality, analyses meet some Bradford Hill criteria.

## Conclusion

The offspring of mothers with postnatal depression are at higher risk of anxiety at 10 years old, while the offspring of mothers with postnatal anxiety are at increased risk of psychotic experiences at 12/13 years old. This study shows that it is possible to identify women whose offspring may be at risk of developing a psychiatric disorder through screening for postnatal depression and anxiety. This may be beneficial for public mental health, to identify at risk groups and the resources needed for early intervention and preventive measures. This holds clinical relevance to the importance of an effective public mental health policy ensuring the provision of support and treatment for maternal mental health disorders throughout the postnatal period, and it may help to reduce offspring risk of mental health difficulties in adolescence. Furthermore, our findings contribute to further understanding the nature of the associations between maternal postnatal psychopathology and offspring mental health in adolescence, suggesting the existence of specific pathways, and thus, further research should disentangle the potential underlying mechanisms of these specific associations. Enhancing understanding of developmental risk factors on subsequent mental illness and the potential mechanisms will improve primary and secondary prevention approaches for at-risk individuals. Here, we suggest some potential genetic, biological and environmental factors that might explain these associations, but further research targeting these mechanisms is required.

## Supplemental Material

sj-docx-1-anp-10.1177_00048674221082519 – Supplemental material for Maternal postnatal depression and anxiety and the risk for mental health disorders in adolescent offspring: Findings from the Avon Longitudinal Study of Parents and Children cohort Supplemental material, sj-docx-1-anp-10.1177_00048674221082519 for Maternal postnatal depression and anxiety and the risk for mental health disorders in adolescent offspring: Findings from the Avon Longitudinal Study of Parents and Children cohort by Isabel Morales-Munoz, Brooklyn Ashdown-Doel, Emily Beazley, Camilla Carr, Cristina Preece and Steven Marwaha in Australian & New Zealand Journal of Psychiatry
