# Supplementary material for: Maternal postnatal depression and anxiety and the risk for mental health disorders in adolescent offspring: Findings from the Avon Longitudinal Study of Parents and Children cohort
Source: Aust N Z J Psychiatry. 2022 Mar 2;57(1):82–92. doi: 10.1177/00048674221082519 (PMC9791327; doi:10.1177/00048674221082519)
Supplement: sj-docx-1-anp-10.1177_00048674221082519 – Supplemental material for Maternal postnatal depression and anxiety and the risk for mental health disorders in adolescent offspring: Findings from the Avon Longitudinal Study of Parents and Children cohort [file sj-docx-1-anp-10.1177_00048674221082519.docx]

**Table S1.** Logistic Regression Analyses between maternal postnatal depression and anxiety, and all the covariates, with adolescent offspring mental health outcomes

|  | **Offspring BPD Symptoms at 11-12 years old** | | | |
| --- | --- | --- | --- | --- |
|  | β | SE | P | OR (95%CI) |
| Postnatal depression at 8 weeks | .001 | .012 | .940 | 1.001 (.978- 1.025) |
| Postnatal anxiety at 8 weeks | .028 | .016 | .079 | 1.028 (.997-1.061) |
| Birth weight, in kg | -.204 | .104 | .051 | .816 (.665-1.001) |
| Child sex | -.011 | .093 | .903 | .989 (.823-1.187) |
| Maternal age at birth | -.007 | .010 | .470 | .993 (.974-1.012) |
| Gestational age | **.067** | **.033** | **.041** | **1.069 (1.003-1.141)** |
| Family adversity index | **.052** | **.011** | **<.001** | **1.054 (1.032-1.076)** |
| Ethnicity | -.081 | .327 | .805 | .923 (.486-1.750) |
|  | **Offspring Depression at 10 years old** | | | |
|  | β | SE | P | OR (95%CI) |
| Postnatal depression at 8 weeks | .003 | .011 | .810 | 1.003 (.981-1.025) |
| Postnatal anxiety at 8 weeks | .029 | .015 | .063 | 1.029 (.998-1.061) |
| Birth weight, in kg | .086 | .101 | .397 | 1.090 (.893-1.329) |
| Child sex | -.019 | .092 | .835 | .981 (.819-1.176) |
| Maternal age at birth | **-.034** | **.010** | **.001** | **.967 (.948-.985)** |
| Gestational age | -.017 | .032 | .604 | .984 (.924-1.047) |
| Family adversity index | **.074** | **.010** | **<.001** | **1.077 (1.057-1.098)** |
| Ethnicity | -.197 | .322 | .541 | .821 (.437-1.543) |
|  | **Offspring Anxiety at 10 years old** | | | |
|  | β | SE | P | OR (95%CI) |
| Postnatal depression at 8 weeks | **.038** | **.017** | **.022** | **1.039 (1.005-1.073)** |
| Postnatal anxiety at 8 weeks | .024 | .023 | .289 | 1.025 (.979- 1.072) |
| Birth weight, in kg | -.177 | .162 | .274 | .838 (.610-1.150) |
| Child sex | -.058 | .147 | .694 | .944 (.780-1.259) |
| Maternal age at birth | .016 | .015 | .298 | 1.016 (.986-1.046) |
| Gestational age | .005 | .050 | .913 | 1.005 (.912-1.108) |
| Family adversity index | **.074** | **.015** | **<.001** | **1.077 (1.046-1.108)** |
| Ethnicity | .006 | .455 | .989 | 1.006 (.412-1.108) |
|  | **Offspring Psychotic Experiences at 12-13 years old** | | | |
|  | β | SE | P | OR (95%CI) |
| Postnatal depression at 8 weeks | .015 | .012 | .225 | 1.015 (.991-1.040) |
| Postnatal anxiety at 8 weeks | **.041** | **.017** | **.016** | **1.042 (1.008- 1.077)** |
| Birth weight, in kg | **-.406** | **.111** | **<.001** | **.667 (.536-.829)** |
| Child sex | **.205** | **.101** | **.042** | **1.227 (1.008-1.495)** |
| Maternal age at birth | -.010 | .011 | .330 | .990 (.969-1.011) |
| Gestational age | **.090** | **.034** | **.009** | **1.094 (1.023-1.170)** |
| Family adversity index | **.044** | **.011** | **<.001** | **1.045 (1.023-1.068)** |
| Ethnicity | .205 | .293 | .484 | 1.227 (.691-2.179) |

*Covariates: Offspring birth weight (kg), offspring gender, age of mother at delivery (years), offspring ethnicity, total Family Adversity Score, and gestational age of offspring at birth

We included both postnatal depression at 8 weeks post-birth and postnatal anxiety at 8 weeks post-birth within the same model.

Abbreviations: BPD, Borderline Personality Disorder; β, Unstandardized beta; SE, Standard Error; P, Statistical significance; OR, Odds Ratio; CI, Confidence Interval

**Table S2.** Direct effects between exposures and outcomes, including covariates, from path analyses

| **Exposure** | **Outcome** | **Estimate** | **P value** |
| --- | --- | --- | --- |
| Maternal postnatal depression | Anxiety at 10y | **0.045** | **<0.001** |
| Maternal postnatal anxiety | Psychotic experiences at 12/13y | **0.031** | **0.028** |
| Sex | Anxiety at 10y | -0.003 | 0.786 |
| Sex | Depression at 10y | 0.004 | 0.700 |
| Sex | BPD symptom at 11/12y | -0.002 | 0.843 |
| Sex | Psychotic experiences at 12/13y | **0.034** | **0.005** |
| Family Adversity Index | Depression at 10y | **0.119** | **<0.001** |
| Family Adversity Index | Psychotic experiences at 12/13y | **0.068** | **<0.001** |
| Family Adversity Index | BPD symptom at 11/12y | **0.095** | **<0.001** |
| Family Adversity Index | Anxiety at 10y | **0.093** | **<0.001** |
| Gestational age | Anxiety at 10y | -0.007 | 0.532 |
| Gestational age | Depression at 10y | **-0.030** | **0.010** |
| Gestational age | BPD symptom at 11/12y | -0.008 | 0.557 |
| Gestational age | Psychotic experiences at 12/13y | **0.031** | **0.013** |
